# *Alicyclobacillus* Contamination in the Production Line of Kiwi Products in China

**DOI:** 10.1371/journal.pone.0067704

**Published:** 2013-07-02

**Authors:** Jiangbo Zhang, Tianli Yue, Yahong Yuan

**Affiliations:** College of Food Science and Engineering, Northwest A&F University, Yangling, PR China; Loyola University Medical Center, United States of America

## Abstract

*Alicyclobacillus* are spoilage microbes of many juice products, but contamination of kiwi products by *Alicyclobacillus* is seldom reported. This study aims to investigate the whole production line of kiwi products in China to assess the potential risk of their contamination. A total of 401 samples from 18 commercial products, 1 processing plant and 16 raw material orchards were tested, and 76 samples were positive, from which 76 strains of microbes were isolated and identified as 4 species of *Alicyclobacillus*, including *Alicyclobacillus acidoterrestris, Alicyclobacillus contaminans*, *Alicyclobacillus herbarius* and *Alicyclobacillus cycloheptanicus*, and another 9 strains as 3 species of *Bacillus* by sequencing of their 16S rDNA. Through phylogenetic tree construction and RAPD-PCR amplification, it was found that there exist genotypic diversities to some extent among these isolates. Four test strains (each from one species of the 4 *Alicyclobacillus* species isolated in this study) could spoil pH adjusted kiwi fruit juice and some commercial kiwi fruit products with producing guaiacol (11–34 ppb).

## Introduction

*Alicyclobacillus* species are a group of thermo-acidophilic, non-pathogenic rod-shaped, endospore-forming bacteria which were first isolated from Japanese hot springs in 1967 and can cause the spoilage of many fruit juice products [1]–[4]. Fruit juice are susceptible to the growth of yeasts, mycelial fungi and lactic acid bacteria due to their ability to grow in high-acid environments [5], but the pH of fruit juice had been thought to be too low for the spore-forming bacteria to grow [6]. In 1982, a large-scale spoilage incident caused by a new type of bacterium happened in Germany [7]. The most common characteristic of the spoilage is a medicinal, antiseptic offensive off-odor in commercial pasteurised apple juice [7]. The microbe causing this spoilage incident was a thermo-acidophilic, endospore-forming bacterium that was later identified as *Alicyclobacillus acidoterrestris* [2]. After this spoilage incident the potential dangers of *Alicyclobacillus* species were realized by the juice industries. During the next decade, after many this kind of thermo-acidophilic bacteria, which were distinct from the bacteria of the genus *Bacillus*, have been reported, they were allocated to a new genus, *Alicyclobacillus*, based on comparative sequence analysis of their 16S rRNA genes and the presence of ω-alicyclic fatty acids in their cell membrane [2]. To date, 19 species, 2 subspecies and 2 genomic species that belong to the genus *Alicyclobacillus* have been identified [8], [9].

The thermal processes to inactivate pathogenic foodborne microorganisms and vegetative non-heat resistant spoilage microorganisms in juice are insufficient to inactivate *Alicyclobacillus* [10]. Chang and Kang [11] also proved that the pasteurisation treatments applied to fruit products are not sufficient to inactivate *Alicyclobacillus* in 2004. A 1998 survey by the European National Food Processors Association showed that 35% of juice manufacturing participants experienced *Alicyclobacillus* spp. related problems. In North America, as well as in Europe, a similar survey in the same year conducted by the Grocery Manufacturers Association of the USA also showed that almost 1/3 of the responded companies had experienced spoilage incidents which may be caused by *Alicyclobacillus* [12]. In 1999, Eiroa et al. [5] found that up to 14.7% of 75 orange juice samples from 11 Brazilian companies to contain *Alicyclobacillus*. According to a survey by the European Fruit Juice Association (AIJN) in 2005, 45% of the 68 companies from the fruit processing industry experienced *Alicyclobacillus* related product contaminations during the three years prior to the survey, including 33% experiencing contaminations more than once [13]. These reports provide evidence to support that the problem caused by *Alicyclobacillus* spp. is a major and widespread microbial spoilage concern for the juice and beverage industries, which has not been thoroughly studied [12], [14].

*Alicyclobacillus* spp. are soil-borne bacteria [15], and there have been reports of *Alicyclobacillus* isolated from orchards [16], [17]. *Alicyclobacillus* have been isolated from many kinds of fruit juices products and concentrated fruit juices products, including apple [18]-[20], pear [16], [17], [14], orange [21], banana [22], watermelon [21], mango [23], lemon [24], grapefruit and blueberry [14] and so on with apple and pear are the most frequent isolation sources, but there are almost no reports about *Alicyclobacillus* contamination of kiwi fruit products. According to a 14-year survey in Argentina, 8556 samples from 7 Argentinean provinces of 19 different kinds of fruit and vegetable juices were analyzed for the presence of *Alicyclobacillus*, and the result showed that there was no *Alicyclobacillus* found in the only one sample of kiwi fruit [25].

Even so, we believe that there might be a potential risk of *Alicyclobacillus* contamination in kiwi fruit products because we have isolated 1 strain of *Alicyclobacillus* from a kiwi fruit product. As the origin source area of kiwi fruit and the largest production country in the world, China’s industry of kiwi fruit products (juice, vinegar, wine, fresh-cut slices) is developing very fast in recent years, and out of 60% of kiwi fruit production are from Shaanxi province [26], especially from Mei county and Zhouzhi county. Gocmen and Pettipher [27] [28] have proved that cell numbers between 10^5^ and 10^6^ CFU/mL of *A. acidoterrestris* produced sufficient guaiacol (2 ppb) to spoil orange and apple juices, but to our knowledge there is still no reports about the growth and taint (mainly guaiacol) production of *Alicyclobacillus* in kiwi fruit juice or commercial beverages related to kiwi fruit, although vanillin, which is a precursor of guaiacol is contained in kiwi fruit. This research aims to investigate the existence condition of *Alicyclobacillus* in the production line of kiwi products (orchards, processing factories and commercial products) in China's Shaanxi province and study the growth to and taint production of *Alicyclobacillus* in kiwi fruit juice and commercial kiwi fruit products to assess the potential risk of *Alicyclobacillus* contamination in kiwi fruit production line under the situation of a rapid development of this industry.

## Materials and Methods

### Sampling and Isolation

**Figure 1:**
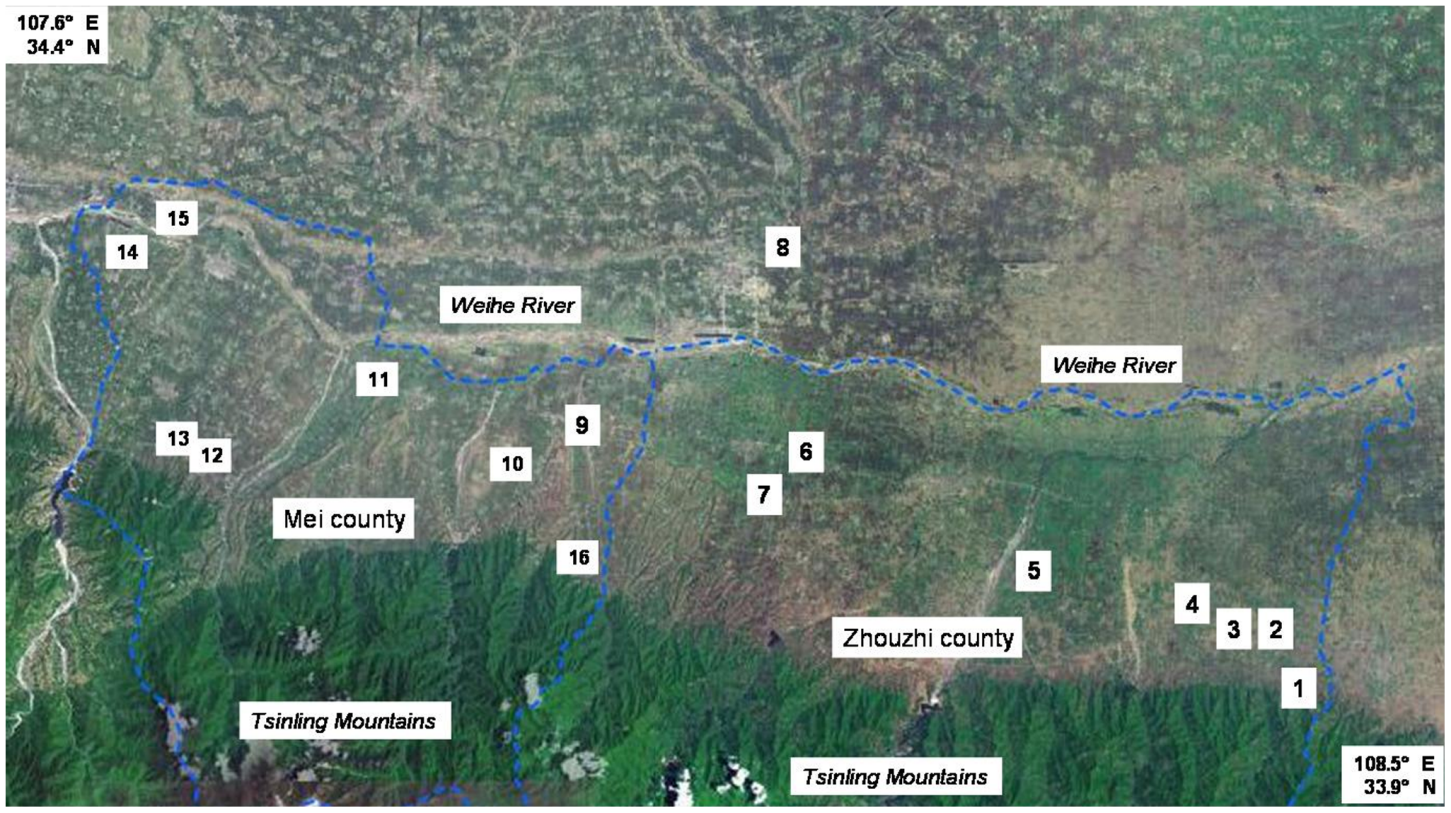
Sampling sites of 16 orchards. The blue dash lines on the map are the administrative boundaries of Mei county and Zhouzhi county which located on the middle of Shaanxi province of China. The plain between the Weihe River and the Tsinling Mountains within these two counties, which is a part of the Guanzhong Plain, is the main region of kiwi fruit production in Shaanxi province.

Samples were collected from three sources: eighteen samples were collected from commercial kiwi products bought from supermarkets, stores in Yangling or online, 102 samples were collected from a Hazard Analysis Critical Control Point accredited fresh-cut and frozen fruit and vegetable producer in Shaanxi province of China (the name of the authority who issued the permission: Baoji DuLe Food Co., Ltd), and 281 samples (fruits, soil and air) were collected from 16 orchards covering main regions of kiwi fruit production in Shaanxi province (Figure 1) (Table 1). The names of the authorities who issued the permissions for each sampling location are also shown in Table 1.

**Table 1:** The number of samples and positive samples of the 16 orchards and their geographic coordinate.

Number of orchards	Locations of orchards	Geographic coordinate of orchards	authority who issued the permission	Number of samples	Positive samples
East					
1	Shaanxi Zhouzhi Bairui Kiwi fruit Experimental Base	108.45°E, 34.05°N	Shaanxi Bairui Kiwi Fruit Research Institute Co. Ltd.	14	5
2	Kiwi fruit Experimental Base of Agricultural Science-technology Demonstration Park (Nanqianhu village in Zhouzhi county)	108.45°E, 34.07°N	the government of Jiufeng township	18	4
3	Gengxi village, Jiufeng township, Zhouzhi county	108.41°E, 34.07°N	p	22	0
4	Liujiabao village, Jixian township, Zhouzhi county	108.38°E, 34.10°N	p	16	4
5	Erhezhuang village, Louguan township, Zhouzhi county	108.27°E, 34.11°N	p	15	6
6	Shangtiantun village, Situn township, Zhouzhi county	108.14°E, 34.17°N	p	19	3
7	Changdong village, Yabai township, Zhouzhi county	108.10°E, 34.15°N	p	14	6
8	Xiajiagou village, Yangcun township, Yangling town	108.11°E, 34.30°N	p	21	7
West					
9	Jinjia village, Hengqu town, Mei county	107.99°E, 34.19°N	p	22	5
10	Tuling village, Tangyu town, Mei county	107.93°E, 34.18°N	p	18	9
11	Taoyuan village, Huaiya town, Mei county(pollution free orchards)	107.85°E, 34.21°N	p	16	3
12	Luoyukou village, Yingtou town, Mei county	107.75°E, 34.16°N	p	8	0
13	Tongyu village, Yingtou town, Mei county	107.74°E, 34.16°N	p	7	2
14	Yuechen village, Diwucun town, Mei county	107.68°E, 34.28°N	p	25	1
15	Chenjiagou village, Diwucun town, Mei county	107.71°E, 34.29°N	p	26	0
16	Kiwi fruit Experimental Station of Northwest A & F University (Qinghua Town)	107.99°E, 34.12°N	Northwest A&F University	20	2
Total				281	57

p: private land, and the owner of the land have given permission to conduct the study on this site.

As the most efficient isolation medium, the *Bacillus acidoterrestris* medium (BAM) [29] which can support nearly all species and all spoilage related species of *Alicyclobacillus*, was used as the medium for the enrichment of *Alicyclobacillus* in this study [9]. A hundred milliliter samples of kiwi products were mixed with 200 mL BAM broth in 500 mL flasks followed by a heat shock treatment at 80°C for 10 min [18] to inhibit the growth of yeast and fungi and promote the germination of *Alicyclobacillus* spores, after which the flasks were shaken at 50°C, 120 rpm in a shaker for 5 days to obtain an enrichment. Then 1 mL of these cultures were diluted appropriately in test tubes and obtain a same heat shock treatment and streaked on BAM plate and yeast starch glucose (YSG) [30] plates duplicately followed by a incubation at 45°C (BAM) or 60°C (YSG) for 4 days. After the enrichment, according to their morphology features, colonies which may be *Alicyclobacillus* species were selected randomly from plates and re-streaked to obtain pure colonies [9]. Then all pure colonies were streaked on pH 7.0 Luria-Bertani (LB) plates. The colonies which did not grow on LB plates were selected and observed with a microscope. Colonies of spore-forming rods were selected for further examination and stored at -40°C in corresponding broth supplemented with 30% sterile glycerol.

The number and the enrichment methods of the samples are shown in Table 2. Samples from the orchards were collected randomly into sterile sample bags from 2 sources: soil and fruits (both on trees and dropped), and the air samples were also collected with BAM plates. The samples were treated immediately after collection with 100 mL sterile water poured into the sample bags directly and then put in a shaker for 10 min at 120 rpm. After settlement the supernatant solution was mixed with 100 mL BAM broth and incubated at 50°C, 120 rpm for 5 days after a heat shock treatment at 80°C for 10 min to obtain an enrichment. The consequent isolation steps of samples from the plant and orchards were the same as the isolation steps of kiwi products samples. The plates containing air samples were incubated at 50°C for 5days directly followed by isolation steps similar to other samples.

**Table 2:** The number and the sampling and enrichment methods of the samples from the producer.

Sample form and sampling sections	Number of samples	Sampling and enrichment methods	Positive samples
**Orchards**			
Kiwi fruits before peeling	10	Samples were put into sterile sample bags and treated immediately after collection with 300 mL sterile water poured into the sample bags and then put in a shaker for 10 min at 120 rpm. After settlement 100 mL of the supernatant solution was mixed with 100 mL BAM broth and incubated at 50°C, 120 rpm for 5 days after a heat shock treatment at 80°C for 10 min to have an enrichment.	2
Kiwi fruits after peeling	12	s	2
Kiwi fruits after color protection treatment	8	s	2
**Washing & fresh-cut shops**			
Kiwi fruits before washing	7	s	1
Kiwi fruits after washing	9	s	1
Kiwi fruit slices	10	s	1
Wash water	5	A total 2500 mL of wash water was collected from 5 sites of the washer randomly with five 500 mL sterile flasks. After delivery to the lab all the samples were mixed with BAM broth at the ratio of 1∶1 in 500 mL flasks and put in a shaker at 50°C, 120 rpm for 5 days after a heat shock treatment at 80°C for 10 min to have an enrichment.	2
Shop environment (walls)	4	Sampling were carried out with sterile cotton bud smearing on the surfaces of the shop environment and then put into test tubes containing 10 mL BAM broth. The tubes were incubated at 50°C for 5 days after a heat shock treatment at 80°C for 10 min as an enrichment.	1
Shop environment (floor)	8	s	2
Shop environment (raw material bins)	9	s	3
Shop environment (air)	10	s	0
**Quick-freezing shop**			
Quick-frozen kiwi fruit slices	10	After melting at room temperature, the samples were treated by the same method as used for samples from the orchards.	1
Total	102		18

s: same as above.

### 16S rRNA Gene Amplification and Sequencing

Selected colonies were grown in corresponding broth, and then their genomic DNA was extracted with the TIANamp Bacteria DNA Kit (TIANGEN, Beijing, China) according to the manufacturer’s instructions. Then a portion of their 16S rRNA gene was amplified using the primers 27F (5′-AGA GTT TGA TCC TGG CTC AG-3′) and 1492R (5′-GGY TAC CTT GTT ACG ACT T-3′) (BGI, supplied by BGI, Beijing, China) [31].

PCR amplifications were performed under conditions as below: fifty microliter of a total reaction volume, 0.3 µL of 5 U/µL *Taq* DNA polymerase (Takara, supplied by Takara Biotechnology Co. Ltd., Dalian, China), 5 µL of 10× PCR reaction buffer, 3 µL of 25 mM MgCl_2_, 4 µL of deoxyribonucleoside triphosphate (dNTPs) mixture (Takara) with 2.5 mM each, 4 µL of each primers, 3 µL of template DNA. PCR reactions were done in an Alpha Unit Block Assembly for Peltier Thermal Cycler DNA Engine Systems (MJ RESEARCH Inc., Watertown, Massachusetts, USA) under conditions as below: initial denaturation at 94°C for 2 min, 30 cycles of denaturation at 94°C for 45 s, annealing at 50°C for 45 s, elongation at 72°C for 2 min, and final elongation at 72°C for 10 min [17].

The PCR products were checked by 1% agarose (Invitrogen, supplied by Invitrogen, Carlsbad, CA92008, USA) electrophoresis with an electrophoresis apparatus (Liuyi, Beijing Liuyi Instrument Factory, Beijing, China) to confirm they contained a 1.5 kb fragment each. Then these fragments were sent to BGI for purification and sequencing.

### Phylogenetic Tree Construction and RAPD-PCR Amplification

The sequence data of 16S rDNA of these isolates were compared to sequences in GenBank using BLAST 2.2.27 and then submitted to GenBank using Sequin. Then these 16S rDNA sequences of isolates were aligned with CLUSTAL W to construct a phylogenetic tree with MEGA 5.10 using both the neighbour-joining method [32] based on the p-distance model and the maximum-parsimony method [33].

RAPD-PCR amplifications were performed in 50 µL volume reactions containing 0.3 µL of 5 U/µL *Taq* DNA polymerase (Takara), 5 µL of 10× PCR reaction buffer, 3 µL of 25 mM MgCl_2_, 4 µL of dNTPs mixture (Takara) with 2.5 mM each, 4 µL of 2 different primers (A-01∶5′-CAGGCCCTTC-3′; AZ-14∶5′-CACGGCTTCC-3′) (BGI). The volume of template DNA was adjusted from 1 to 4 µL to get the clearest electrophoresis patterns. The PCR conditions were the same as above, except for annealing at 38°C for 45 s, and elongation at 72°C for 2 min 30 s. The results were checked by 1% agarose electrophoresis.

### Enumeration of Growth and GC-MS Analysis for Taint Production

Four strains (C-ZJB-12-32, C-ZJB-12-35, C-ZJB-12-55 and C-ZJB-12-69, each 1 strain for one species of *Alicyclobacillus* isolated in this study) were selected and inoculated into BAM broth and incubated for 12 h to reach the log phase for activation, then these cultures were serially diluted (to make the number of CFU/mL of each sample was below 100 after inoculation) and each inoculated into 150 mL kiwi fruit juice (pH adjusted to 4.2 using 4 M NaOH) and 150 mL kiwi fruit juice (pH 2.5, without adjustment), then all 8 samples were incubated at 45°C for 21 days. Activated C-ZJB-12-35 was also inoculated into 150 mL of some commercial kiwi products mentioned in the sampling and isolation part, including 2 kinds of kiwi fruit juice (pH 3.5, soluble solids (SS) content 10°Brix, raw kiwi fruit juice content 60%), vinegar (pH 2.5, 5°Brix) and wine (pH 3.5, 7°Brix, alcohol 6%) (<100 CFU/mL after inoculation) and incubated at 45°C for 21 days. All 14 samples were plated onto BAM agar every day for the first week of incubation and every 3 days for the remainder of the experiment. Samples of kiwi fruit juice without pH adjustment obtained another test with a heat shock treatment of 80°C for 10 min before plating to check spores in them.

According to some previous reports [27] [28] [34] [35], guaiacol and 2 kinds of halophenols, 2,6-dibromophenol (2,6-DBP) and 2,6-dichlorophenol (2,6-DCP) were chosen as aim taint compounds. Standard solutions were prepared with guaiacol (Fluka Analytical, 2931 Soldier Springs Road, Laramie, WY, USA), 2,6-DBP (SUPELCO, 595 North Harrison Road, Bellefonte, PA, USA) and 2,6-DCP (SIGMA-ALDRICH, Co., 3050 Spruce Street, St. Louis MO, USA) in concentrations of 2.5, 5,7.5, 10 and 20 ppb in distilled water. A 5 ppb standard solution for guaiacol was also prepared in kiwi fruit juice as a contrast. All standard solutions were kept at 4°C in the dark until analysis (within 2 days). Ten milliliter of samples which could accumulate >10^6^ CFU/mL after incubation and all standard solutions were transferred to 20 mL glass vials. After a 15-min equilibrium at 45°C, a solid phase microextraction (SPME) fiber (50/30mm DVB/Carboxen/PDMS; Supelco Co., Bellefonte, PA, USA) was exposed to the headspace of the vials at 45°C for 1 h to extract their volatile compounds.

After the extraction, the fiber was inserted into a Thermo-Finnigan Trace GC ultra/Trace DSQ (Thermo-Finnigan, San Jose, CA, USA) injection port using a 30 m ×0.25 mm i.d.×0.25 µm DB-Wax column (Agilent, Palo Alto, CA, USA). The GC-MS conditions were as follows: carrier gas He at 1 ml/min, splitless mode, injector temperature 250°C, starting temperature 50°C (2 min), final temperature 230°C (5 min), temperature rate 10°C /min, ion source temperature 230°C, scanning mass range 50–350 m/z. NIST 2002 fragmentation spectra database was used for identifications.

### Nucleotide Sequence Accession Numbers

**Figure 2:**
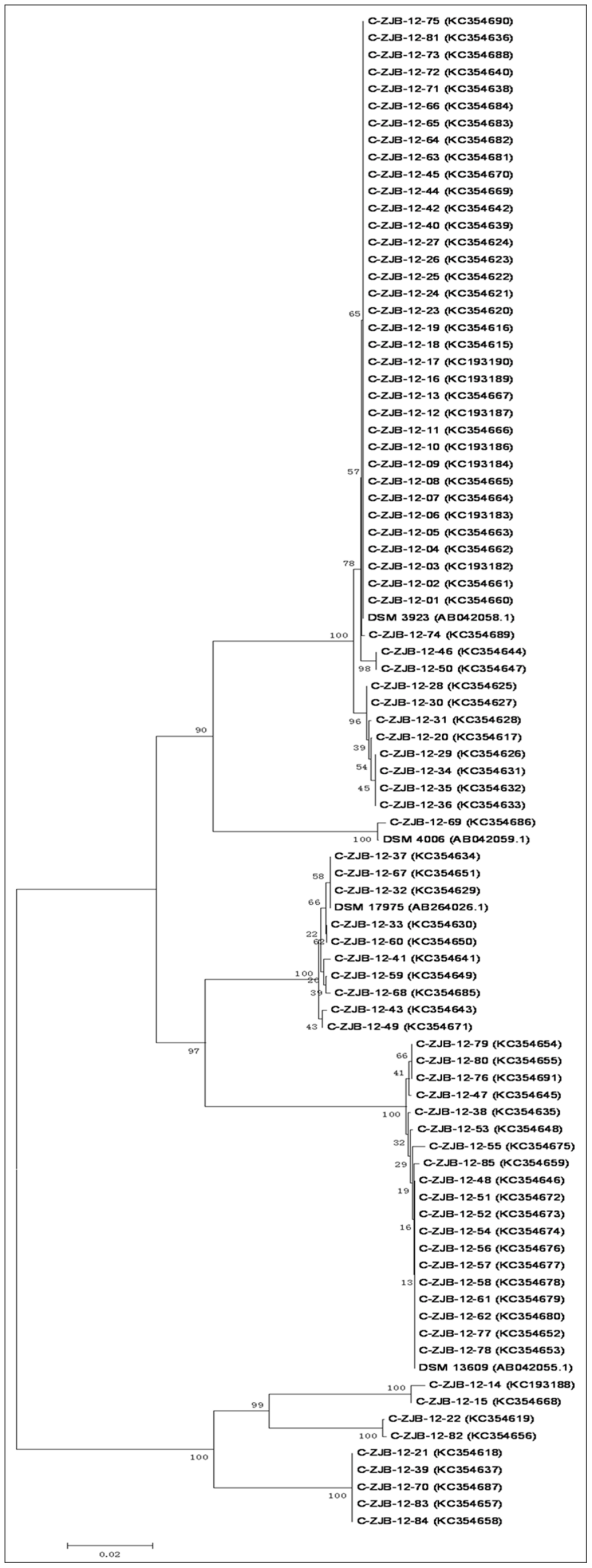
Neighbour-joining phylogenetic tree of isolates and reference species. The 4 reference species are *Alicyclobacillus acidoterrestris* DSM 3923, *Alicyclobacillus cycloheptanicus* DSM 4006, *Alicyclobacillus contaminans* DSM 17975, and *Alicyclobacillus herbarius* DSM 13609. Construction is based on 16S rRNA gene sequence comparisons. Bootstrap percentages based on 1000 replicates are shown. Bar, p-distance (0.02 substitutions per nucleotide position).

All the 16S rRNA gene (16S rDNA) sequences were deposited in GenBank (accession numbers KC193182 to KC193190 and KC354615 to KC354691, also see details in Figure 2).

## Results

### Positive Percentages and Properties of Isolates

A total of 401 samples were tested, and 76 samples were positive (*Alicyclobacillus* detected, 19.0%), from which 85 strains of microbes were isolated and identified. The total percentages of positive samples of the 16 orchards and all sampling sections of the producer were 20.3% and 17.6%, respectively (Table 1; Table 2), and only one sample (around 140 CFU/mL) among 18 commercial kiwi fruit products was detected as positive (5%), from which the one isolate was identified as *Alicyclobacillus acidoterrestris* later.

All isolates were Gram-positive, endospore-forming rods, and could form creamy white or yellowish, opaque colonies. Some isolates were motile. Among all 85 isolates, 1 was isolated from commercial products, 16 were isolated from the producer and 68 were isolated from the 16 orchards, including 26 from fruits samples (mainly from drop fruits), 37 from soil samples and 5 from air samples. The data revealed that *Alicyclobacillus* are soil-borne bacteria, as reported by Deinhard et al. in 1987 [15]. All samples from orchards’ air were negative except 2 air samples (formed 5, 7 colonies on plates respectively) from the orchard in Xiajiagou village, which might be because of the sprinkle on the day of sampling.

### Identification of Isolates

As shown in Table 3, the similarity (max identity of BLAST results) of 16S rDNA among all isolates was beyond 99% except C-ZJB-12-85, and the number of nucleotides sequenced of all isolates were beyond 1400 (almost complete sequence of 16S rDNA) except C-ZJB-12-53 and C-ZJB-12-55. These mean that the isolates were very likely the same species as their nearest phylogenetic neighbours showed in Table 3. Forty six isolates from 20 sources (all sampling sections of the quick-frozen kiwi fruit slice producer and 7 orchards: the orchard in Xiajiagou village, Bairui Kiwi fruit Experimental Base, Changdong village, Liujiabao village, Kiwi fruit Experimental Station of Northwest A & F University, kiwi fruit Experimental Base of Agricultural Science-technology Demonstration Park, and Yuechen village) of total 85 isolates (54.1%) were identified as *Alicyclobacillus acidoterrestris* through the sequence data compared with nucleotides sequences in GenBank, which means that *Alicyclobacillus acidoterrestris* is the most widespread species of *Alicyclobacillus*.

**Table 3:** All isolated strains, their sources, number of nucleotides sequenced, nearest phylogenetic neighbour and similarity.

Isolated strains	Source	No. of nucleotides sequenced	Nearest phylogenetic neighbour (GenBank accession number)	Similarity
C-ZJB-12-01	Kiwi fruit wine (pH 3.5)	1423	*Alicyclobacillus acidoterrestris* (NR_040844.1)	99.9%
C-ZJB-12-02	Kiwi fruits before peeling	1413	*Alicyclobacillus acidoterrestris* (NR_040844.1)	99.8%
C-ZJB-12-03	s	1424	*Alicyclobacillus acidoterrestris* (AB682390.1)	99.6%
C-ZJB-12-04	s	1437	*Alicyclobacillus acidoterrestris* (AJ133631.1)	99.7%
C-ZJB-12-05	s	1428	*Alicyclobacillus acidoterrestris* (NR_040844.1)	99.8%
C-ZJB-12-06	Kiwi fruits after peeling	1428	*Alicyclobacillus acidoterrestris* (NR_040844.1)	99.6%
C-ZJB-12-07	Kiwi fruits after color protection treatment	1430	*Alicyclobacillus acidoterrestris* (NR_040844.1)	99.6%
C-ZJB-12-08	Kiwi fruits before washing	1430	*Alicyclobacillus acidoterrestris* (NR_040844.1)	99.6%
C-ZJB-12-09	s	1433	*Alicyclobacillus acidoterrestris* (NR_040844.1)	99.6%
C-ZJB-12-10	Kiwi fruits after washing	1438	*Alicyclobacillus acidoterrestris* (NR_040844.1)	99.6%
C-ZJB-12-11	Wash water	1432	*Alicyclobacillus acidoterrestris* (NR_040844.1)	99.7%
C-ZJB-12-12	Shop environment (walls)	1440	*Alicyclobacillus acidoterrestris* (AB682390.1)	99.6%
C-ZJB-12-13	Shop environment (floor)	1422	*Alicyclobacillus acidoterrestris* (AB682390.1)	99.9%
C-ZJB-12-14	Shop environment (raw material bins)	1470	*Bacillus coagulans* (AB696800.1)	98.8%
C-ZJB-12-15	s	1441	*Bacillus coagulans* (AB696800.1)	99.9%
C-ZJB-12-16	s	1441	*Alicyclobacillus acidoterrestris* (NR_040844.1)	99.2%
C-ZJB-12-17	s	1435	*Alicyclobacillus acidoterrestris* (NR_040844.1)	99.5%
C-ZJB-12-18	Fruits from the orchard in Xiajiagou village	1409	*Alicyclobacillus acidoterrestris* (NR_040844.1)	99.9%
C-ZJB-12-19	s	1434	*Alicyclobacillus acidoterrestris* (NR_040844.1)	99.9%
C-ZJB-12-20	s	1412	*Alicyclobacillus acidoterrestris* (AB059675.1)	99.8%
C-ZJB-12-21	Soil from the orchard in Xiajiagou village	1450	*Bacillus fumarioli* (AJ581126.1)	99.7%
C-ZJB-12-22	s	1429	*Bacillus ginsengihumi* (NR_041378.1)	99.9%
C-ZJB-12-23	Air from the orchard in Xiajiagou village	1429	*Alicyclobacillus acidoterrestris* (NR_040844.1)	99.7%
C-ZJB-12-24	s	1422	*Alicyclobacillus acidoterrestris* (NR_040844.1)	99.8%
C-ZJB-12-25	s	1432	*Alicyclobacillus acidoterrestris* (NR_040844.1)	99.6%
C-ZJB-12-26	s	1409	*Alicyclobacillus acidoterrestris* (NR_040844.1)	99.8%
C-ZJB-12-27	s	1435	*Alicyclobacillus acidoterrestris* (NR_040844.1)	99.6%
C-ZJB-12-28	Fruits from the orchard in Bairui Kiwi fruit Experimental Base	1423	*Alicyclobacillus acidoterrestris* (AB059675.1)	99.5%
C-ZJB-12-29	s	1417	*Alicyclobacillus acidoterrestris* (AB059675.1)	99.6%
C-ZJB-12-30	s	1419	*Alicyclobacillus acidoterrestris* (AB059675.1)	99.5%
C-ZJB-12-31	s	1423	*Alicyclobacillus acidoterrestris* (AB682383.1)	99.4%
C-ZJB-12-32	s	1430	*Alicyclobacillus contaminans* (NR_041475.1)	99.3%
C-ZJB-12-33	s	1418	*Alicyclobacillus contaminans* (AB264027.1)	99.4%
C-ZJB-12-34	s	1429	*Alicyclobacillus acidoterrestris* (AB059675.1)	99.6%
C-ZJB-12-35	s	1412	*Alicyclobacillus acidoterrestris* (AB059675.1)	99.6%
C-ZJB-12-36	s	1430	*Alicyclobacillus acidoterrestris* (AB059675.1)	99.5%
C-ZJB-12-37	s	1431	*Alicyclobacillus contaminans* (NR_041475.1)	99.5%
C-ZJB-12-38	Soil from the orchard in Bairui Kiwi fruit Experimental Base	1444	*Alicyclobacillus herbarius* (AB681266.1)	99.2%
C-ZJB-12-39	s	1433	*Bacillus fumarioli* (J581126.1)	99.8%
C-ZJB-12-40	s	1428	*Alicyclobacillus acidoterrestris* (NR_040844.1)	99.9%
C-ZJB-12-41	s	1451	*Alicyclobacillus contaminans* (AB264027.1)	99.2%
C-ZJB-12-42	Fruits from the orchard in Changdong village	1412	*Alicyclobacillus acidoterrestris* (NR_040844.1)	99.9%
C-ZJB-12-43	s	1437	*Alicyclobacillus contaminans* (AB264027.1)	99.5%
C-ZJB-12-44	s	1422	*Alicyclobacillus acidoterrestris* (NR_040844.1)	99.6%
C-ZJB-12-45	s	1424	*Alicyclobacillus acidoterrestris* (NR_040844.1)	99.7%
C-ZJB-12-46	s	1451	*Alicyclobacillus acidoterrestris* (AB682390.1)	99.9%
C-ZJB-12-47	s	1455	*Alicyclobacillus herbarius* (AB681266.1)	99.2%
C-ZJB-12-48	s	1453	*Alicyclobacillus herbarius* (AB681266.1)	99.5%
C-ZJB-12-49	s	1437	*Alicyclobacillus contaminans* (AB264027.1)	99.6%
C-ZJB-12-50	s	1438	*Alicyclobacillus acidoterrestris* (AB682390.1)	99.8%
C-ZJB-12-51	Soil from the orchard in Changdong village	1449	*Alicyclobacillus herbarius* (AB681266.1)	99.5%
C-ZJB-12-52	s	1426	*Alicyclobacillus herbarius* (AB681266.1)	99.4%
C-ZJB-12-53	s	993	*Alicyclobacillus herbarius* (AB681266.1)	99.7%
C-ZJB-12-54	s	1448	*Alicyclobacillus herbarius* (AB681266.1)	99.1%
C-ZJB-12-55	s	1003	*Alicyclobacillus herbarius* (AB681266.1)	99.0%
C-ZJB-12-56	s	1439	*Alicyclobacillus herbarius* (AB681266.1)	99.2%
C-ZJB-12-57	s	1429	*Alicyclobacillus herbarius* (AB681266.1)	99.4%
C-ZJB-12-58	s	1435	*Alicyclobacillus herbarius* (AB681266.1)	99.2%
C-ZJB-12-59	Soil from the orchard in Liujiabao village	1401	*Alicyclobacillus contaminans* (AB264027.1)	99.5%
C-ZJB-12-60	s	1439	*Alicyclobacillus contaminans* (AB264027.1)	99.0%
C-ZJB-12-61	s	1448	*Alicyclobacillus herbarius* (AB681266.1)	99.5%
C-ZJB-12-62	s	1449	*Alicyclobacillus herbarius* (AB681266.1)	99.2%
C-ZJB-12-63	s	1433	*Alicyclobacillus acidoterrestris* (NR_040844.1)	99.8%
C-ZJB-12-64	s	1426	*Alicyclobacillus acidoterrestris* (NR_040844.1)	99.5%
C-ZJB-12-65	s	1429	*Alicyclobacillus acidoterrestris* (NR_040844.1)	99.7%
C-ZJB-12-66	s	1431	*Alicyclobacillus acidoterrestris* (AB682390.1)	99.6%
C-ZJB-12-67	Fruits from the orchard in Shangtiantun village	1440	*Alicyclobacillus contaminans* (NR_041475.1)	99.2%
C-ZJB-12-68	s	1452	*Alicyclobacillus contaminans* (AB264027.1)	99.5%
C-ZJB-12-69	s	1435	*Alicyclobacillus cycloheptanicus* (AB680830.1)	99.4%
C-ZJB-12-70	Soil from the orchard in Shangtiantun village	1442	*Bacillus fumarioli* (AJ581126.1)	99.9%
C-ZJB-12-71	Soil from the orchard in Kiwi fruit Experimental Station of Northwest A & F University	1422	*Alicyclobacillus acidoterrestris* (NR_040844.1)	99.8%
C-ZJB-12-72	s	1412	*Alicyclobacillus acidoterrestris* (NR_040844.1)	99.9%
C-ZJB-12-73	Fruits from the orchard in kiwi fruit Experimental Base of Agricultural Science-technology Demonstration Park	1434	*Alicyclobacillus acidoterrestris* (AB682390.1)	99.7%
C-ZJB-12-74	Soil from the orchard in kiwi fruit Experimental Base of Agricultural Science-technology Demonstration Park	1407	*Alicyclobacillus acidoterrestris* (NR_040844.1)	99.9%
C-ZJB-12-75	s	1428	*Alicyclobacillus acidoterrestris* (NR_040844.1)	99.6%
C-ZJB-12-76	s	1430	*Alicyclobacillus herbarius* (AB681266.1)	99.2%
C-ZJB-12-77	s	1452	*Alicyclobacillus herbarius* (AB681266.1)	99.3%
C-ZJB-12-78	s	1456	*Alicyclobacillus herbarius* (AB681266.1)	99.2%
C-ZJB-12-79	s	1451	*Alicyclobacillus herbarius* (AB681266.1)	99.0%
C-ZJB-12-80	s	1451	*Alicyclobacillus herbarius* (AB681266.1)	99.3%
C-ZJB-12-81	Soil from the orchard in Yuechen village	1420	*Alicyclobacillus acidoterrestris* (NR_040844.1)	99.7%
C-ZJB-12-82	Soil from the orchard in Erhezhuang village	1460	*Bacillus ginsengihumi* (FJ357590.1)	100%
C-ZJB-12-83	s	1459	*Bacillus fumarioli* (AJ581126.1)	99.9%
C-ZJB-12-84	s	1457	*Bacillus fumarioli* (AJ581126.1)	99.8%
C-ZJB-12-85	Soil from the orchard in Tongyu village	1447	*Alicyclobacillus herbarius* (AB681266.1)	98.8%

s: same as above.

In this study, 10 strains of *Alicyclobacillus contaminans* (4 orchards: the orchards in Bairui Kiwi fruit Experimental Base, Changdong village, Liujiabao village, and Shangtiantun village), 19 strains of *Alicyclobacillus herbarius* (5 orchards: the orchards in Bairui Kiwi fruit Experimental Base, Changdong village, Liujiabao village, kiwi fruit Experimental Base of Agricultural Science-technology Demonstration Park, and Tongyu village) and 1 strains of *Alicyclobacillus cycloheptanicus* (Shangtiantun village) were also isolated.

One thing to be noted was that there were 9 strains of thermo-acidophilic *Bacillus* isolated, including 2 strains of *Bacillus coagulans* from shop environment (raw material bins), 5 strains of *Bacillus fumarioli* from soil of the orchards in 4 locations (Xiajiagou village, Shangtiantun village, Bairui Kiwi fruit Experimental Base, Erhezhuang village), and 2 strains of *Bacillus ginsengihumi* from soil of the orchard in Erhezhuang village and Xiajiagou village.

### Phylogenetic Positions and Genotypic Diversities of Isolates

From the neighbour-joining phylogenetic tree of the 85 isolates and 4 reference species (Figure 2) we can see that all strains fell into 7 clusters. All the strains of each of the 7 species (4 *Alicyclobacillus* species and 3 *Bacillus* species) clustered together. The biggest cluster (46 isolate strains and 1 reference strain) including all *Alicyclobacillus acidoterrestris* strains were mainly composed of 2 groups. The bootstrap values of the branches within these 2 groups and the *Alicyclobacillus contaminans* cluster and the *Alicyclobacillus herbarius* cluster were low (some bootstrap values were even below 30), which indicated that the differences among these strains were very small according to their 16S rDNA sequences.

**Figure 3:**
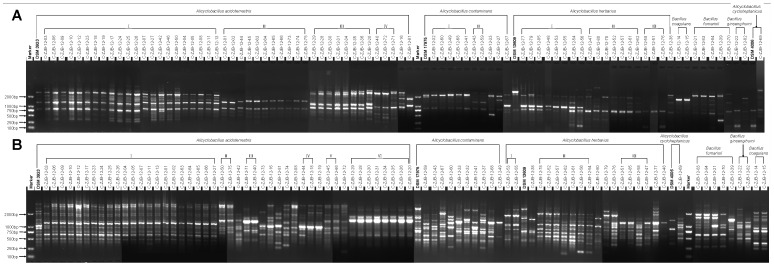
Electrophoresis patterns of RAPD-PCR of the genomic DNA of isolates and reference species. The 4 reference species are *Alicyclobacillus acidoterrestris* DSM 3923, *Alicyclobacillus cycloheptanicus* DSM 4006, *Alicyclobacillus contaminans* DSM 17975, *Alicyclobacillus herbarius* DSM 13609. I–VI indicate groups within a certain species. Marker, DNA marker DL2000 (BGI). A, Primer A-01; B, Primer AZ-14.

Genotypic diversities of all 85 isolates and 4 reference species were evaluated by RAPD-PCR (Figure 3). Generally, the RAPD-PCR with primer AZ-14 (pattern B in Figure 3) generated more bands after electrophoresis than with primer A-01 (pattern A in Figure 3), which might be caused by the relatively higher affinity of primer AZ-14 to the genomic DNA of *Alicyclobacillus*. Therefore, the genotypic diversity revealed through RAPD-PCR with primer AZ-14 was bigger than with primer A-01. In detail, in pattern A, within the cluster of *Alicyclobacillus acidoterrestris*, there were 4 groups and 2 unique strains which were different from each other, as well as from any strains of the 4 groups. Whereas in pattern B, there were 6 groups and 7 unique strains in the same cluster. But the biggest 2 groups in this cluster were similar. Group I was composed of 22 strains in both pattern A and B, and 16 strains of them were the same, while group III in pattern A and group VI in pattern B were composed of exactly the same 8 strains, which agreed with the result of the phylogenetic tree’s corresponding part precisely. Within the cluster *Alicyclobacillus contaminans* there were 2 groups and 3 unique strains in pattern A, but each strain including the reference strain DSM 17975 belong to this cluster in pattern B was different from each other, which suggested there might exist great genotypic diversity within this species of *Alicyclobacillus* in the kiwi fruit production area in Shaanxi province of China. Although there were 3 groups within the cluster *Alicyclobacillus herbarius* in both 2 patterns, but pattern B still showed bigger genotypic diversity, with 6 unique strains, whereas there was only 1 unique strain in pattern A. But the 2 patterns also shared some similarity. There were 2, 5, and 2 same strains between group I, II and III, respectively.

### Growth and Taint Production

**Figure 4:**
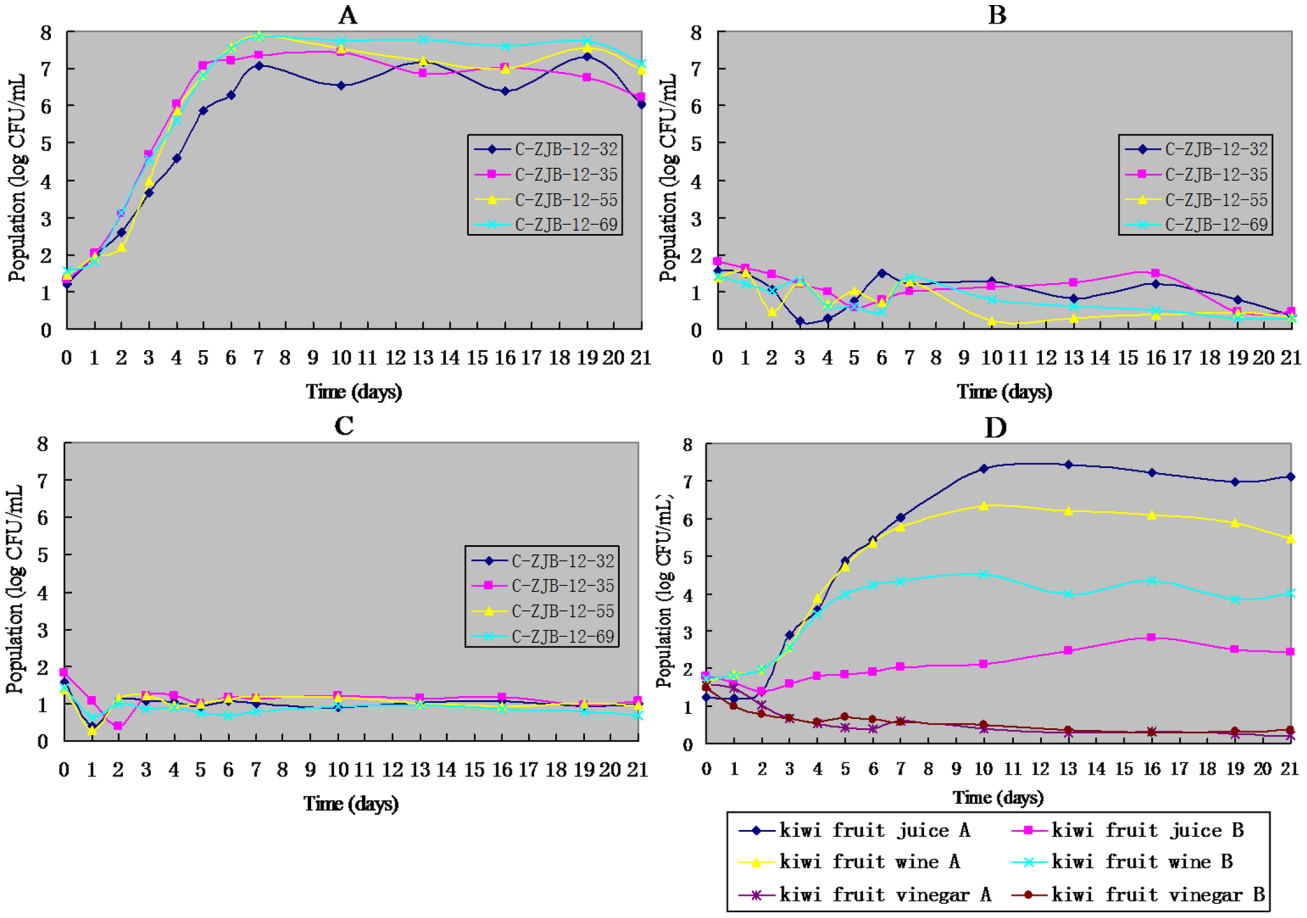
Changes in microbial populations during the 45°C incubation. A, pH adjusted kiwi fruit juice inoculated with 4 strains of *Alicyclobacillus*; B, kiwi fruit juice inoculated with 4 strains of *Alicyclobacillus*; C, kiwi fruit juice inoculated with 4 strains of *Alicyclobacillus* (with a heat shock treatment); D, six kinds commercial kiwi fruit products inoculated with C-ZJB-12-35.G.

Changes in microbial populations over incubation are shown in Figure 4. Replicate uninoculated control samples of 2 kinds of kiwi fruit juice and 6 commercial kiwi fruit products had undetectable *Alicyclobacillus* populations (<30 CFU/mL) over incubation (data not shown). Microbial population levels of all pH adjusted kiwi fruit juice samples reached >10^7^ CFU/mL within 7 days of incubation, and the growth curves of all 4 test strains were similar. All curves in Figure 4B did not seem extending regularly, which might be caused by the testing errors (with a inoculation volume of 100 µL, there usually were only <5 CFUs on a plate). But it was obvious that there was hardly any growth in all kiwi fruit juice samples without pH adjustment. In all these samples a certain number of spores kept their abilities of reproduction throughout the whole incubation time (Figure 4C). One juice sample of the six commercial kiwi fruit products samples reached population levels >10^7^ CFU/mL within 10 days, and the 2 wine samples accumulated *Alicyclobacillus* population levels 10^6^ and 10^4^, respectively. *Alicyclobacillus* did not grow obviously in the rest samples of this group.

**Figure 5:**
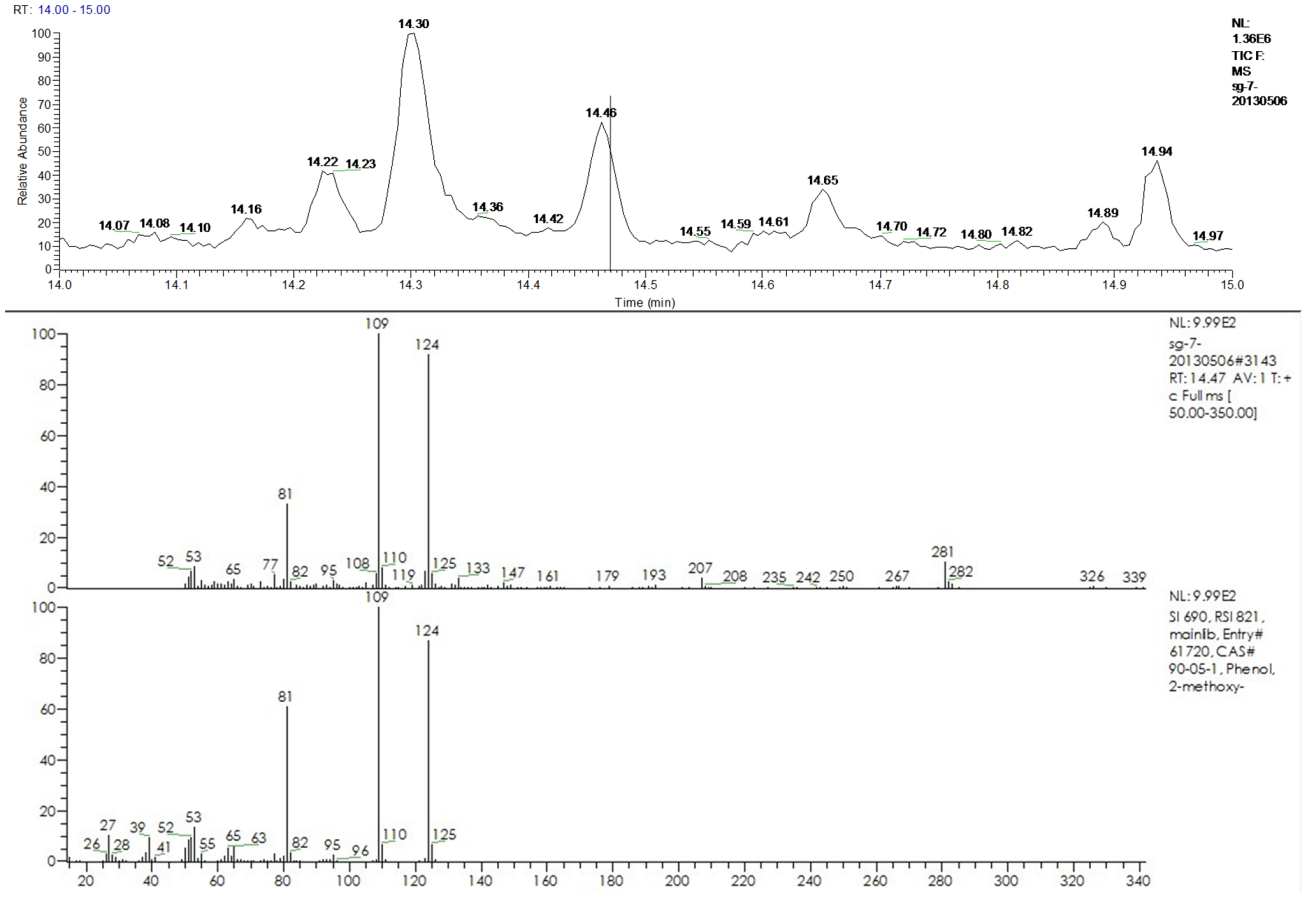
A GC–MS chromatogram of pH adjusted kiwi fruit juice inoculated with C-ZJB-12-35. Mass spectrums from the peak (14.47 min) thought to be guaiacol in this sample and from standard guaiacol are also shown. The compound peaked at 281 m/z may come from the SPME fiber, because it exist in the peak around 14.47min in all samples. Because the scanning mass range was 50–350 m/z, peaks below 50 m/z were not included. Both of these may reduce the SI and RSI values.

**Figure 6:**
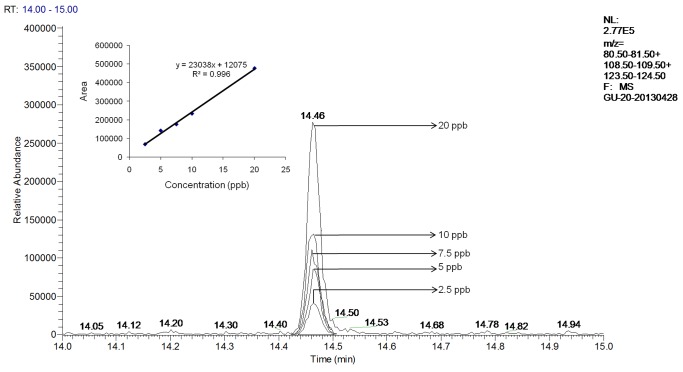
A GC–MS chromatogram of all standard solutions and a standard curve generated by Excel. For comparison purposes only peaks between the mass range of 81, 109, 124 m/z are displayed.

A GC–MS chromatogram, a mass spectrum from the peak thought to be guaiacol in the sample of pH adjusted kiwi fruit juice inoculated with C-ZJB-12-35 and a mass spectrum of standard guaiacol are shown in Figure 5. The compound peaked at 14.47 min should be guaiacol based on the values of SI and RSI given by the NIST 2002 library and comparison with the retention time of standard solutions. Three major mass fragments of guaiacol (mass to charge, m/z, 81,109, 124) can be seen in Figure 5. The GC–MS chromatograms of other chosen samples were similar to the above one, according to which guaiacol were produced by all chosen samples. No 2,6-DBP and 2,6-DCP were detected. A GC–MS chromatogram of all standard solutions and a standard curve generated by Excel are shown in Figure 6. The mass spectrums of the peaks at 14.47 min in the TIC chromatograms of the contrast 5 ppb standard solution for guaiacol in kiwi fruit juice and the sample of commercial kiwi fruit wine A show low SI and RSI values of many compounds through NIST library. The reason might be there were several compounds detected at this retention time or the limit of the instrument precision, and changing a more suitable column or optimizing the conditions of microextraction and GC-MS might obtain better results. The peak areas of guaiacol in samples and the calculated concentrations of guaiacol are shown in Table 4 (see Chromatogram S1-S6 for their raw data files). According to the standard curve all the samples accumulated guaiacol between 11–34 ppb after the 21 days incubation.

**Table 4:** The peak areas of guaiacol of samples and the calculated concentrations.

Samples	Start Retention Time (min)	End Retention Time (min)	Peak area	Concentration (ppb)
C-ZJB-12-32 in A	14.44	14.54	280389.50	11.65
C-ZJB-12-35 in A	14.44	14.53	435151.81	18.36
C-ZJB-12-55 in A	14.43	14.52	678012.37	28.91
C-ZJB-12-69 in A	14.44	14.50	812313.79	34.74
C-ZJB-12-35 in B	14.43	14.50	650602.09	27.72

According to the standard curve, concentrations were calculated based on the peak areas (mass range: 81, 109, 124 m/z).

A: pH adjusted kiwi fruit juice; B: commercial kiwi fruit juice A.

## Discussion

The decline of percentages of positive samples from orchards to producer shops and commercial products reveals the distribution rule of microbes in a whole production line. There seem no clear relations among the percentages of positive samples of the 16 orchards, and the percentages of some orchards (Gengxi village, Luoyukou village, Chenjiagou village) are even 0%, despite their number of samples. This may be because of the sampling locations. Positive samples are likely from drop fruits and the soil around them (data not shown). Therefore, there are probably other species of *Alicyclobacillus* except the 4 species found in this study because of the randomness and limited quantity of sampling.

The result that *Alicyclobacillus acidoterrestris* is the most widespread species of *Alicyclobacillus* in this study is similar to many previous reports [36], [17], [14]. *Alicyclobacillus acidoterrestris* is the most important spoilage microbe of juice products and off-odour (mainly guaiacol) producer [18], [37], therefore there will be a potential risk of spoilage when more and more kiwi fruits from these orchards are used as raw materials for juice, wine and other products in the future. Except 2 isolates, all the isolates from all sampling sections of the producer, including the raw material orchard, processing shops and final products, are identified as *Alicyclobacillus acidoterrestris*. This result indicates that the potential risk mentioned above truly exists, which is also proved by the fact that the only one isolate from commercial kiwi fruit products is identified as *Alicyclobacillus acidoterrestris* too. If more producers’ shops are tested, other species of *Alicyclobacillus* may be found.

To our knowledge, this is the first report on the isolation of *Alicyclobacillus contaminans* and *Alicyclobacillus herbarius* from China’s orchards. *Alicyclobacillus herbarius* and *Alicyclobacillus cycloheptanicus* has also been proved as taint producers [27] [36]. Therefore, kiwi fruit products coming from these orchards may be contaminated by these non-*acidoterrestris Alicyclobacillus*.

*Alicyclobacillus acidoterrestris* and *Alicyclobacillus contaminans* were widely distributed among various fruit orchards in Japan [36]. According to the results of this study, it is thought that *Alicyclobacillus acidoterrestris, Alicyclobacillus contaminans,* and *Alicyclobacillus herbarius* might be the predominant species of *Alicyclobacillus* in kiwi fruit orchards in Shaanxi province of China.

The D_80°C_ of *Bacillus coagulans* is 40 min in a double concentrated tomato paste media according to Sandoval et al. [38], and *Bacillus fumarioli* grows optimally at pH 5.5 and 50°C [39], while *Bacillus ginsengihumi* can also grow in acid media at temperature up to 50°C [40]. These reports proved that these *Bacillus* can be an interference to the detection of *Alicyclobacillus* from samples and cause false positive results, although there are no reports relating them with spoilage of juice products and guaiacol production.

Through the analysis of the 2 patterns it was thought the division of genotypic diversities among a relatively large number of samples (as in this study) though PAPD-PCR with different primers may be different because of the different affinity between the primer and the sample strain’s genomic DNA. There seems no large possibility of RAPD-PCR as a traceability method of *Alicyclobacillus* contamination, because the RAPD-PCR results showed that one certain genotypic group within a cluster of a certain species could be isolated from different sources (such as group I of the cluster of *Alicyclobacillus acidoterrestris,* which included strains from 3 sources in pattern A and 4 sources in pattern B, with the plant of the producer of frozen kiwi fruit slice as one source), and strains from one source could be divided into different groups within a cluster of a certain species (such as strains from Xiajiagou village [C-ZJB-12-18 ∼ C-ZJB-12-27] which belonged to *Alicyclobacillus acidoterrestris* were in 2 groups in pattern A and 3 groups in pattern B). These results show some differences from Groenewald’s [17] report.

From Figure 4A,B and C we can see that the main reason for *Alicyclobacillus* to unable to grow in raw kiwi fruit juice is not its containing some bacteriostatic factors, but its relatively low pH (2.5) compared with other fruit juices (apple and orange, pH 3.5–4.0), and their spores can retain viability in raw kiwi fruit juice and grow and produce taint like in apple and orange juices when the pH rises while dilution or mixing with other relatively high pH juices to make palatable commercial products. Figure 4D proves this conclusion further. The growth curve in the commercial kiwi fruit juice A products are similar to the ones in pH adjusted kiwi fruit juice, only showing longer lag phase and longer time (about 8–10 days) to reach the population levels of 10^7^ CFU/mL, which may be because of the lower pH and different media contents compared with pH adjusted kiwi fruit juice. The reason why C-ZJB-12–35 is unable to grow in the 2 vinegar samples may lie in their low pH (2.5). The alcohol in the 2 wine samples might have negative influence on the growth of *Alicyclobacillus*, but according to the GC-MS chromatogram of wine A, a small amount of guaiacol should exist in the sample, although guaiacol might not be separated well with other compounds. As for another commercial juice which unable to support the growth of C-ZJB-12-35, it is inferred that it does not contain enough raw kiwi fruit juice as described on its packaging (no antiseptics found).

Guaiacol has been found in all 5 samples proved that it is the most important taint compound, which is similar to Pettipher’s report [28]. It has been proved that the best estimated threshold (BET) value for odour of guaiacol was around 2 ppb in apple juice [28], and similar threshold values for odour of guaiacol in apple juice were even lower in other reports [41] [42]. Although there are still no reports about such values in kiwi fruit juice, it is thought they might not be much higher than in apple juice, therefore the guaiacol levels in this study are high enough to make a consumer aware of the spoilage. The different amount of guaiacol production among the 5 samples might be attributed to the differences among species, cell numbers. Goto et al. [36] has proved *Alicyclobacillus herbarius* to produce guaiacol, although in the same study, they did not detect guaiacol from *Alicyclobacillus contaminans*, which may be because of the limit of the test method and the culture media. In this study, we first proved that *Alicyclobacillus contaminans* could produce guaiacol (11.65 ppb) in pH adjusted kiwi fruit juice. There were no halophenols (2,6-DBP and 2,6-DCP) detected in all samples in this study, although *Alicyclobacillus acidoterrestris* has been proved to produce guaiacol and 2,6-DBP, and *Alicyclobacillus cycloheptanicus* has been proved to produce guaiacol and both 2,6-DBP and 2,6-DCP in Gocmen’s study [27]. This might be attributed to the media used in this study.

Although there was only one *Alicyclobacillus* strain isolated from commercial kiwi fruit products, *Alicyclobacillus* strains were isolated from kiwi fruit product processing plant and raw material orchards, and there existed genotypic diversities to some extent. Furthermore, the test strains from all 4 species isolated in this study can spoil the pH adjusted kiwi fruit juice and some commercial products, and their spores can retain viability in raw kiwi fruit juice. Therefore, there do exist a potential risk of *Alicyclobacillus* contamination, even spoilage incidents, when a large quantity of kiwi fruits were used as raw materials to make juice or wine or other products, although there is no report about such incidents yet in China. Because of the similarity of 16S rDNA between one species and its subspecies could beyond 99% in genus *Alicyclobacillus* (e.g. between some strains of *Alicyclobacillus acidocaldarius* and *Alicyclobacillus acidocaldarius* subsp. *Rittmannii* after searching through NCBI), the genotypic diversities (through RAPD-PCR) of these isolates indicate that there might be some new subspecies among them. Therefore, further research will be focused on the growth conditions and off-odour production of all isolated *Alicyclobacillus* in kiwi fruit juice and commercial products to further assess their capability of contamination, and looking for new subspecies to expand our knowledge about *Alicyclobacillus*.

## Supporting Information

Chromatogram S1 **The GC-MS raw data file of pH adjusted kiwi fruit juice inoculated with C-ZJB-12-32.**
(RAW)

Chromatogram S2 **The GC-MS raw data file of pH adjusted kiwi fruit juice inoculated with C-ZJB-12-35.**
(RAW)

Chromatogram S3 **The GC-MS raw data file of pH adjusted kiwi fruit juice inoculated with C-ZJB-12-55.**
(RAW)

Chromatogram S4 **The GC-MS raw data file of pH adjusted kiwi fruit juice inoculated with C-ZJB-12-69.**
(RAW)

Chromatogram S5 **The GC-MS raw data file of** **commercial kiwi fruit juice A inoculated with C-ZJB-12-35.**
(RAW)

Chromatogram S6 **The GC-MS raw data file of commercial kiwi fruit wine A inoculated with C-ZJB-12-35.**
(RAW)

Chromatogram S7 **The GC-MS raw data file of 5-ppb standard solution for guaiacol in kiwi fruit juice.**
(RAW)
